# Carlsberg alibi marketing in the UEFA euro 2016 football finals: implications of *Probably* inappropriate alcohol advertising

**DOI:** 10.1186/s12889-018-5449-y

**Published:** 2018-04-25

**Authors:** Rachael Murray, Magdalena Opazo Breton, John Britton, Jo Cranwell, Bruce Grant-Braham

**Affiliations:** 1 UK Centre for Tobacco & Alcohol Studies, Faculty of Medicine & Health Sciences, University of Nottingham, Nottingham, UK; 2 UK Centre for Tobacco and Alcohol Studies, Department for Health, University of Bath, Bath, UK; 3 School of Sports and Physical Activity, Faculty of Management, Bournemouth University, Poole, Dorset UK

**Keywords:** Alcohol, Advertising, Alibi, Exposure, Impressions, Children

## Abstract

**Background:**

Alcohol advertising is a key driver of alcohol consumption, and is prohibited in France by the Loi Evin. In 2016 the Danish brewer *Carlsberg* sponsored the UEFA Euro 2016 finals, held in France, and used the alibis ‘*Probably’* and ‘*…the best in the world’* in place of *Carlsberg* in pitch-side advertising. We have quantified the advertising exposure achieved during the final seven games in the UEFA Euro 2016 championship.

**Methods:**

Appearances of the Carlsberg alibis ‘*Probably’* and ‘*the best in the world*’ were counted and timed to the nearest second during all active play in live coverage of quarter final, semi-final and final matches broadcast in the UK. We used census data and viewing figures from Kantar Media to estimate gross and per capita impressions of these advertisements in the UK population.

**Results:**

In 796 min, 29 s of active play there were 746 alibi appearances, totalling 68 min 35 s duration and representing 8.6% of active playing time. Appearances were particularly frequent at the end of normal time, extra time and penalties. The seven matches delivered up to 7.43 billion Carlsberg alibi impressions to UK adults and 163.3 million to children. In the only match involving a second country with laws prohibiting alcohol advertising (France versus Iceland), exposure occurred for only 1.8% of playing time.

**Conclusions:**

Alibi marketing achieved significant advertising coverage during the final seven EURO 2016 championship games, particularly to children. Since ‘*Probably*’ is registered by Carlsberg as a wordmark this advertising appears to contravene the Loi Evin, though Carlsberg have defended their marketing actions.

**Electronic supplementary material:**

The online version of this article (10.1186/s12889-018-5449-y) contains supplementary material, which is available to authorized users.

## Background

Alcohol consumption is a growing threat to global health. The World Health Organisation (WHO) estimates that in 2012 alcohol consumption caused 3.3 million deaths, nearly 6% of the global total, with the loss of 139 million disability-adjusted life years (DALYs) [1]. These figures are rising quickly: mortality has increased by over 80%, and DALYs lost by more than twofold, since 2001 [2]. Alcohol product marketing, which includes promotion through sponsorship and other links to national and transnational sporting activities, is a driver of alcohol consumption identified by the WHO as a serious concern [3]. Indeed, a recent systematic review of 12 longitudinal studies found that all reported significant associations between exposure to, awareness of, engagement with and/or receptivity to alcohol marketing at baseline and initiation of alcohol use, initiation of binge drinking, drinking in the previous 30 days and/or alcohol problems at follow up in youth populations [4]. For this reason, television advertising of alcohol is now subject to controls in many countries [1], including many European Union Member States [1]. In France the statutory legislation addressing the marketing and advertising of alcohol products is the *Code de la Santé Publique (*Code of Public Health) which incorporates the Loi Evin (Law No. 91–32 of 10 January 1991). The purpose of the Loi Evin is to protect public health [5]. The Loi Evin places a total ban on the direct or indirect advertising of all alcoholic beverages over 1.2% ABV on television and also prohibits sponsorship of sport events by alcohol companies. Further, it specifically forbids the targeting of minors [6].

This is not the first time Carlsberg has been implicated in Loi Evin controversy. in 2016 its packaging was challenged and found to transgress the restrictions laid down in Articles L. 3323–2 and L. 3323–4 of the French Public Health Code [7]. As a result, Carlsberg was ordered to withdraw the packaging from sale. In 2014 the Jury of Advertising Ethics investigated Carlsberg’s Facebook pages in response to complaints by the National Association of Prevention in Alcohol and Addiction, though these complaints were not fully upheld [8].

In summer 2016, France hosted the 2016 Union of European Football Associations (UEFA) European Championship. Carlsberg Group, the fourth largest international brewery company in the world and operating in more than 150 markets [9] was a sponsor of the event. During transmission of the group stage matches of the tournament it became evident that Carlsberg may have adopted alibi marketing methods (whereby core elements of a brand’s identity, such as a strapline, word, colour or shape, are used in advertising instead of the brand’s name or logo) in order to advertise their brand at the Championship.

In the UEFA Euro 2016 broadcasts the usual *Carlsberg* brand name and trademark was entirely replaced on digital advertising billboards surrounding the football pitch by two alibis: the word *‘Probably’*, and the phrase *‘… the best in the world’*. A recent report documents that on average, more than 100 alcohol marketing references were broadcast (including pitch side advertising, branded merchandise, television advertisements, sponsor lead-ins and branded packaging) in a selection of 18 matches across the UK, France and Ireland [10]. To further quantify the extent of this practice, and to estimate the advertising exposure gained in the form of gross impressions, we recorded and have quantified Carlsberg alibi exposure during the last seven European Championship games broadcast in the United Kingdom (UK).

## Methods

We descriptively studied alcohol content and estimated exposure in the final stage of UEFA Euro 2016. Live coverage of the UEFA quarter final, semi-final and final matches, held in France between June 30th and July 10th 2016 and broadcast in the UK was recorded in its entirety (Table 1). Our coding periods included any time of active play in the game, from kick-off to final whistle in the first and second halves of standard and extra time, and from the point the ball was placed for the first penalty to the scoring of the last penalty in matches that were settled by penalty shoot-out. Our coding instrument separately listed each appearance of ‘*Probably’* and ‘*the best in the world*’ displayed in the characteristic *Carlsberg* font on a green background on digital advertising billboards along the perimeter of the pitch. For each appearance the time started and time ended, in minutes and seconds (i.e., 6:30–6:54) by match period (first half, second half, extra time and penalties) was recorded. Visual occurrences of the word ‘*Probably’* or phrase ‘*the best in the world*’ that appeared in clear, uninterrupted view on the screen received a single count in each instance. The duration of each visual occurrence was timed to the nearest second. All this information was recorded in a separate Excel files for each match along with general information about the match (start time, time ended, teams playing, date, stage in the championship). To ensure the accuracy and reliability of coding, the TV coverage for two of the seven games was coded independently by two coders (RM and JC) using the play, pause, review method previously reported [11, 12] and any differences resolved by discussion. UK viewing figures for the UK were supplied by Kantar Media.

**Table 1:** Characteristics of last seven UEFA 2016 European Championship matches broadcast on UK television

	Poland v Portugal	Wales v Belgium	Germany v Italy	France v Iceland	Portugal v Wales	Germany v France	Portugal v France
Date	30-Jun-16	01-Jul-16	02-Jul-16	03-Jul-16	06-Jul-16	07-Jul-16	10-Jul-16
Kick off time	20.00	20.00	20.00	20.00	20.00	20.00	20.00
Tournament stage	Quarter final	Quarter final	Quarter final	Quarter final	Semi final	Semi final	Final
Channel	ITV	BBC	BBC	ITV	ITV	BBC	BBC & ITV
Active playing time (sec)	7514	5725	8165	5550	5581	7568	7686
% Viewing (18+ yrs)*	10%	10%	18%	11%	12%	17%	21%
% Viewing (4–17 yrs) ^†^	5%	5%	9%	5%	5%	10%	11%

*Total population for adults 18 years and older is 59.1million people

^†^Total population for children 4 to 17 years old is 10.3 million people

To study exposure to alcohol content we analysed the distribution of Carlsberg alibi appearances by type of visual occurrence (‘*Probably’* or ‘*the best in the world*’) and used that distribution to compute cumulative gross and per capita impressions, as has been previously reported [13, 14]. To generate the cumulative distributions of Carlsberg alibi appearances by match and type of visual occurrence, we disaggregated the data on total duration of each visual occurrence to second-by-second observations by match period. We coded each second-by-second observation with a ‘1’ if it contained the Carlsberg alibi visual occurrence or ‘0’ if they did not. For each type visual occurrence, we then computed the cumulative frequencies per 5-min-interval for each period of each match. Appearances that overlapped between intervals were coded in both intervals. We combined these distributions with viewing figures for the UK territory and with UK mid-year population estimates [15] to obtain cumulative gross and per capita impressions for children (4 to 17 years old) and adults (18 years and above) in the UK for each match period and for each match.

The study used publicly available television broadcasts and did not involve any human participants, and therefore did not require ethics approval.

## Results

The seven football matches studied were transmitted between the 30th June and 10th July 2016. Four matches were broadcast in the UK by the British Broadcasting Corporation (BBC) and four by the Independent Television Network (ITV) (the final was broadcast simultaneously on BBC and ITV). The broadcasts included a total of 47,779 s (796 min, 29 s) of active play and each game was viewed by between 10% and 21% of the adult population (18 years old and above), and between 5% and 11% of children aged 4 to 17 years (Table 1).

Our coding identified a total of 746 appearances of the two logos, of which 614 (82%) were of ‘*Probably*’ and 132 (18%) ‘*the best in the world*’. ‘*Probably*’ imagery was present for a total of 3133 s (52 min, 13 s; 6.6% of total active play time), and ‘*the best in the world*’ for 982 s (16 min, 22 s; 2.1% of total active play time), with a combined total of 4115 s (68 min, 35 s (8.6% of total active play time). Of the combined appearances and duration of the two logos, ‘*Probably*’ imagery accounted for 82.3% and 76.7%, respectively. The number of appearances of both alibis varied substantially between games, and was particularly low in France versus Iceland quarter final game (Table 2). The percentage of active playing time in which these logos were visible varied substantially between games, from 1.8% in the France-Iceland quarter-final to 13.4% in the Germany-Italy quarter-final (Table 2).

**Table 2:** Number and duration of appearances of ‘probably’ and ‘the best in the world’ imagery by match (seconds)

	France v Portugal	Germany v France	Portugal v Wales	France v Iceland	Germany v Italy	Wales v Belgium	Poland v Portugal	TOTAL
Total number ‘Probably’ appearances	108	64	58	13	190	58	123	614
Total number ‘The best in the world’ appearances	23	20	20	0	24	14	31	132
Total number alibi branding appearances	131	84	78	13	214	72	154	746
Total duration ‘Probably’ appearances	594	305	195	99	897	376	667	3133
Total duration ‘The best in the world’ appearances	199	134	121	0	199	117	212	982
Total duration alibi branding appearances	793	439	416	99	1096	493	879	4115
% playing time where alibi branding appearances occur	10.3	5.8	7.5	1.8	13.4	8.6	11.7	8.6

**Fig. 1:**
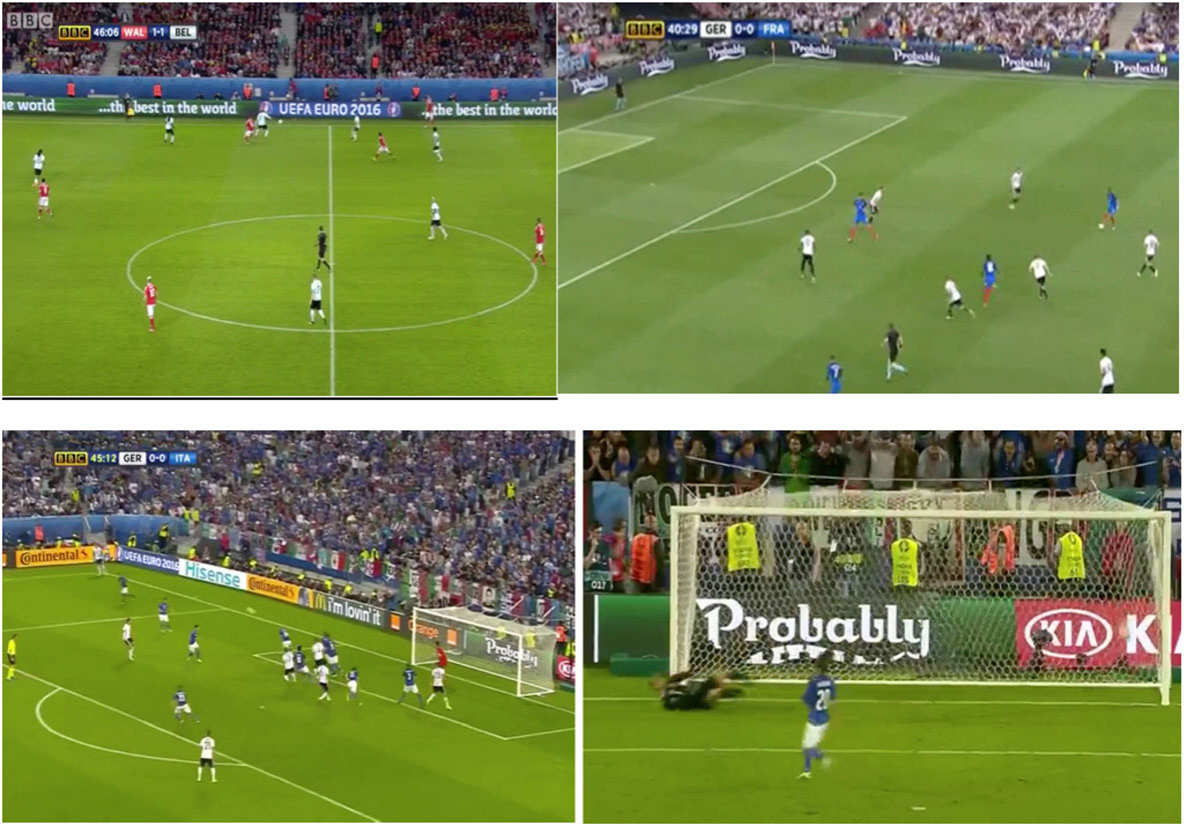
Screenshots of alibi appearances

The majority of logo appearances (406, totalling 2198 s/36 min, 38 s duration) occurred on pitch sideline billboards only; there were 86 appearances (totalling 374 s (6 min, 14 s) duration) behind the goal line only; and 254 (1643 s/27 min, 23 s duration) appearances simultaneously on sidelines and behind goal lines. Of the two logos, ‘*Probably’* appeared relatively frequently behind the goal line (Table 3, Fig. 1).

**Table 3:** Location, frequency and duration (seconds) of ‘*probably*’ and ‘*the best in the world*’ imagery across the final seven UEFA 2016 European Championship matches

	Probably’	The best in the world’	Total
Total number of sideline appearances	359	47	406
Total number of goal line appearances	79	7	86
Total number of simultaneous side and goal line appearances	176	78	254
Overall total number of appearances	614	132	746
% total	82.3%	17.7%	
Total duration of sideline appearances	1872	326	2198
Total duration of goal line appearances	336	38	374
Total duration of simultaneous side and goal line appearances	1025	618	1643
Overall total duration of appearances	3233	982	4215
% total	76.7%	23.3%

**Fig. 2:**
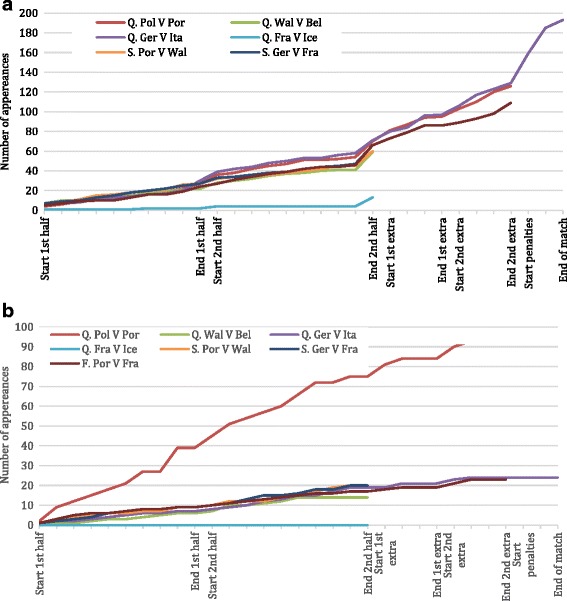
**a** Cumulative Alcohol Appearances by 5-min-interval for *`Probably’.* **b** Cumulative Alcohol Appearances by 5-min-interval for *`the best in the world’*

The frequency of appearances of both ‘*Probably*’ and ‘*the best in the world*’ increased progressively during the first and most of the second halves of matches (Fig. 2a and b respectively), but for ‘*Probably*’ increased during the last few minutes of the second half. For matches that went to extra time, impressions of ‘*Probably*’ then occurred at a higher rate during extra time, and this rate increased still further during penalties (Fig. 2a).

The number of alcohol content impressions delivered within individual matches also varied widely; assuming matches were viewed in their entirety, the lowest exposure was evident in the France versus Iceland game, 92.3 million impressions to the adult population aged 18 or above (1.5 per capita) and 6.9 million impressions to children aged 4 to 17 years old (0.7 per capita); whilst the highest exposure occurred during the Germany versus Italy match, 2.1 billion impressions to the adult population (34.3 per capita) and 163.3 million impressions to children (15.5 per capita). In total, up to 7.43 billion and 358.6 million Carlsberg alibi impressions were delivered to adults and children respectively across the seven matches viewed (Table 4. A more detailed breakdown of impression distribution within and across matches can be seen in Additional file 1: Tables S1 and S2, available in online supplement).

**Table 4:** Gross and Per Capita Impressions^*^ of Cumulative Alcohol Appearances by Type, Match Period, Population Group and Match

				Gross Impressions (in millions)						Per Capita					
	Match	Alcohol content	Pop. Group	End of 1st half	End of 2nd half	End of 1st extra	End of 2nd extra	End of penalties	Total (any alc.)	End of 1st half	End of 2nd half	End of 1st extra	End of 2nd extra	End of penalties	Total (any alc.)
Quarter final	Poland v Portugal	*Probably*	18+	168.8	437.5	593.8	787.6	–	981.4	2.7	7.1	9.6	12.7	–	15.9
			4–17	12.7	33.3	45.0	60.2		75.4	1.2	3.2	4.3	5.7	–	7.1
		*The best in the world*	18+	81.3	156.3	175	193.8	–	981.4	1.3	2.5	2.8	3.1	–	15.9
			4–17	6.4	12.2	13.7	15.2		75.4	0.6	1.2	1.3	1.4	–	7.1
	Wales v Belgium	*Probably*	18+	250.7	672.4	–	–	–	829.3	4.1	10.9	–	–	–	13.4
			4–17	20.4	53.8	–	–	–	71.3	1.9	5.1	–	–	–	6.7
		*The best in the world*	18+	68.4	156.9	–	–	–	829.3	1.1	2.6	–	–	–	13.4
			4–17	5.6	13.0	–	–	–	71.3	0.5	1.2	–	–	–	6.7
	Germany v Italy	*Probably*	18+	292.3	691.9	945.3	1257.1	1880.8	2114.7	4.7	11.2	15.3	20.3	30.4	34.2
			4–17	22.1	53.4	72.5	97.0	145.0	163.3	2.1	5.1	6.9	9.2	13.7	15.5
		*The best in the world*	18+	68.2	185.2	204.6	233.9	233.9	2114.7	1.1	3	3.3	3.8	3.8	34.2
			4–17	5.3	14.5	16.0	18.3	18.3	163.3	0.5	1.4	1.5	1.7	1.7	15.5
	France v Iceland	*Probably*	18+	14.2	92.3	–	–	–	92.3	0.2	1.5	–	–	–	1.5
			4–17	1.1	6.9	–	–	–	6.9	0.1	0.7	–	–	–	0.7
		*The best in the world*	18+	0	0	–	–	–	92.3	0	0	–	–	–	1.5
			4–17	0	0	–	–	–	6.9	0	0	–	–	–	0.7
Semi final	Portugal v Wales	*Probably*	18+	196.8	682.7	–	–	–	828.5	4.5	11	–	–	–	13.4
			4–17	13.9	31.0	–	–	–	41.7	1.3	2.9	–	–	–	3.9
		*The best in the world*	18+	65.5	145.8	–	–	–	828.5	1.1	2.4	–	–	–	13.4
			4–17	4.8	10.7	–	–	–	41.7	0.5	1.0	–	–	–	3.9
	Germany v France	*Probably*	18+	279.3	682.7	–	–	–	889.6	4.5	11	–	–	–	14.4
			4–17	26.5	64.3	–	–	–	84.4	2.5	6.1	–	–	–	8.0
		*The best in the world*	18+	93.1	206.9	–	–	–	889.6	1.5	3.3	–	–	–	14.4
			4–17	9.0	20.1	–	–	–	84.4	0.9	1.9	–	–	–	8.0
Final	Portugal v France	*Probably*	18+	308.5	848.2	1105.3	1400.9	–	1696.5	5	13.7	17.9	22.6	–	27.4
			4–17	27.0	74.4	96.9	121.7	–	147.6	2.6	7.0	9.2	11.5	–	14.0
		*The best in the world*	18+	115.7	218.5	244.2	295.6	–	1696.5	1.9	3.5	3.9	4.8	–	27.4
			4–17	10.1	19.2	21.4	25.9	–	147.6	1.0	1.8	2.0	2.5	–	14.0

^*^Calculations are based on Mid-year population estimates for the UK, which is 10.6 million people for population 4 to 17 years old, and 61.9 million for people over 18 years old

## Discussion

This study demonstrates that despite a national prohibition of television advertising of alcohol in France under the *Loi Evin*, Carlsberg achieved over 70 min and 746 separate instances of apparent promotion of its branding and therefore, potentially, its beer through the use of two alibis: *‘Probably’* and *‘… the best in the world’* during the last seven games of the UEFA 2016 European Championships. Given the television viewing audience for each of these games, this advertising translated into between 92.3 million and 2.1 billion impressions of any alcohol content to the adult population, and between 6.9 and 163.3 million alcohol impressions to children aged 4 to 17 years old, although the distribution of these impressions varied both within and between the seven games investigated. Our figures for the seven games provide confirmation that an earlier estimated high exposure to these logos during the final match [16] also applied in the knock-out games; and by inference also occurred during the group stage matches.

The anticipated TV audience for UEFA Euro 2016 in 230 territories around the world was high: 150 million spectators were expected to follow each game live [17]. The demographics of that audience have not been made public at the time of writing but if the international audience profile for the UEFA Euro 2016 follows that of the UK then an estimated 12.9 million children were exposed to Carlsberg billboards (based on 8.6% of the TV audience being aged 17 or under). An average of 829,000 children were exposed to Carlsberg alibi branding in the UK; if TV viewing figures in France mirror those of the Brazil World Cup, this translates to an average of 387,000 children exposed in each game. This exposure has occurred despite Loi Elvin prohibition of the targeting of minors. The explanation for the much lower alibi content in the France versus Iceland than the other matches we coded is not clear, but it is noteworthy that Iceland has its own laws prohibiting alcohol advertising [18], suggesting that alcohol advertising may have been reduced in that match to achieve relative compliance with Icelandic law. Iceland’s “Afengislog” (Law on Alcohol) clearly and simply states that “all advertising and marketing [of alcohol] is banned” and where television is concerned there is a “ban on advertising of alcohol and unique brands” [19].

Carlsberg has sponsored football clubs and tournaments including eight European championship finals, since at least 1988 [20]. In 2016, Carlsberg anticipated that the Western European beer market would be static, apart from, “*some positive impact during the early summer from UEFA EURO 2016*™” [9]. Carlsberg activated its sponsorship of UEFA EURO 2016™ planning for it to be *“be an important event for the brand”* [9] as football sponsorship had become an integral element of its commercial activity. Carlsberg has described its relationship with football previously as *“a great fit”* [21], being “*part of Carlsberg’s DNA”* [22] and “*a key pillar of the Carlsberg brand, both short and longer term*” [23].

Carlsberg’s traditional trademark logos are based on an original hand-drawn design by Thorvald Bindesbøll in 1904 [24, 25] (Fig. 1) but marketing has been extended to include other words and phrases often sharing the same font and general appearance. The phrase *‘Carlsberg - probably the best lager in the world’* was registered as a word mark in Europe in the year 2000 [26], and has been used by Carlsberg in a range of advertisements applying the phrase to a range of settings, establishing both the word ‘Probably’ and *‘… the best …. in the world’* as brand alibis. ‘*Probably’* was registered as a European wordmark in 2010. Carlsberg’s *“Probably the best lager in the world*” word mark has been described as an example of one which *“acts as a direct carrier of the brand’s equity reminding consumers of their liking for the brand and reinforcing the brand equity at repeated exposures”* [27]*.* Since beer is a heavily advertised and competitive product category, market advantages are derived from relatively small product differences, creating a greater reliance on communicative platforms. Butler and Berry used *‘Carlsberg—probably the best lager in the world’* as an example of a positive brand claim in the form of a slogan [28]. Such slogans are intended to affect how consumers perceive a brand, both in its own right and when judged against the competition, by creating brand awareness by linking the brand to a product category; shaping brand evaluations by priming specific brand associations and transferring likeability; and reinforcing brand awareness and evaluations by serving as a memory aid [29].

Previous research has shown that alibi marketing has been used to circumvent restrictions on sponsorship of Formula One racing by Philip Morris International, through their use of ‘barcode’ designs as a substitute for *Marlboro* logos after the European Directive on tobacco advertising came into force in 2005 [30]. The alibi logos, which were not registered trademarks, were in due course voluntarily withdrawn. However in the present study we demonstrate a perhaps more egregious example of advertising through the use of alibis, which in this case are registered trademarks [31, 32]. By simply displaying the word *“Probably”* and the slogan *“The best beer in the world,”* shortened to *“….the best in the world”,* Carlsberg have been credited with having solved, from its point of view, the problem of the Loi Evin, *“in a very creative way by simply using the slogan with which the company advertised its products from 1973 to 2011 worldwide”* [33]. Carlsberg has been described as one of *“the big winners of Euro2016 with Probably”* [34]*,* and it has been hypothesized that the “marques alibis” had successfully worked around the Loi Evin whilst drawing attention to the process by which the subtle messages linking the alibi trademark and the mother brand had been correlated in the minds of consumers [35]. Indeed, Glendinning (2016) calculated that Carlsberg had achieved a successful 50% prompted recall by using its *‘Probably’* slogan as an ‘alibi brand’ on the stadium LED boards throughout the UEFA EURO2016 tournament [36].

Despite being regarded as some of the strictest laws on alcohol advertising in Europe [37] the French *Loi Evin* has been variously described as controversial [38], ineffective [39] and its policing has been criticised by its very creator, Claude Evin [40]. The Loi Evin has been consistently challenged, particularly by the alcohol industry [38] and as a result has been modified. Politicians including Emmanuel Macron, now the French President, was one such proponent. Whilst he personally failed, the law was subsequently changed in a way seen as being favourable to French regional wine producers [41] and future public health-motivated law makers must resist such influential lobbies.

Section L3323–3 of the French Public Health Code specifically bans the use of, *“Propaganda or advertising”…. “in favour of an organisation, service, activity, product or article other than an alcoholic beverage which, by its design, use of a name, trademark, advertising emblem or other distinctive sign, recalls an alcoholic beverage”* [41]. *We suggest that Carlsberg’s “Probably” message was not only a design, but a registered trademark, an advertising emblem and a distinctive sign that recalled an alcoholic beverage and therefore it contravened the Code*. Some restrictions imposed by the Loi Evin have been lifted since 1991, including the use of billboards in sports grounds for alcohol advertising, however the ban on television transmission restrains this advertising for major events. [42], and this research therefore demonstrates an apparent contravention of the Loi Evin. Despite this Carlsberg defended their marketing actions et the Euro 2016 championships, reportedly stating that they “applied their own strict marketing standards in addition to legal requirements in countries where we operate” [43]. It is the author’s suggestion that the ban in all sports grounds should be re-imposed.

This study is subject to a number of limitations. We were unable to measure the effect of exposure on use of alcohol in our study, however there is strong evidence that exposure to such imagery in other media increases alcohol consumption. Calculation of gross and per capital impressions assumed that the measured audience were present and viewing matches for the entire broadcast. We did not code matches from the earlier stages of the tournament, or advertisements within broadcasts, where present. Finally, our coding only allowed for estimation of exposure in the television broadcast and thus our estimates are applicable to the television viewership; in future it would be interesting to code exposure to spectators within the football stadia to gain estimates of the scale of the problem to those attending live sporting events.

Our findings suggest that Iceland, a country with a relatively clear and simple ‘Afengislog’ has demonstrated a positive influence in reducing exposure of minors to alcohol advertising at UEFA Euro 2016 and other countries could learn from this experience in attempt to draft legislation which will avoid such circumvention. Future lawmakers also need to be aware of the arguments being used when alcohol producers are promoting their low or alcohol-free products. These often share the same branding as the producer’s full alcohol product and therefore the non-alcoholic products are providing an alibi. In addition, some alcohol producers are associating their advertising with responsible drinking alibi messages which contain alcohol product trademarks. For example, in an open letter to Jean Todt (FIA president) from Mariann Skar, Secretary General in the European Alcohol Policy Alliance, and supported by 40 public health and civil society organisations from around the world, Heineken’s 5-year F1 sponsorship is heavily criticised for, “linking a popular motor sport to a significant cause of avoidable physical, mental and social harm and more specifically one of the major killers on our roads, drink driving” [44]. This global campaign incorporates Heineken’s characteristic red star and green branding on billboards which also have a prominent “When You Drive, Never Drink” message (http://www.eurocare.org/media_centre/press_releases/formula_1_puts_heineken_in_the_driving_seat). Indeed Heineken has said that it will use F1 to promote this campaign, supported by ambassador Sir Jackie Stewart [45]. In both situations potential consumers, and particularly impressionable youth, might well not be sufficiently sophisticated to tell the difference. Future legislation needs to recognise this to enable minors to be protected.

## Conclusion

Given our estimate that up to 358.6 million alcohol impressions were delivered to children aged four to 17 years old during the final seven matches of the UEFA EURO 2016 championship, in apparent contravention of the Loi Evin, it is imperative that steps are taken to eliminate this avenue of advertising from future events, and indeed as a general advertising strategy. Further consideration also needs to be given as to how best regulate other forms of potential alibi marketing, such as non-alcoholic versions of alcoholic drinks.

## Additional file

Additional file 1: **Table S1.** Gross and Per Capita Impressions of Cumulative Carlsberg Alibi Appearances by Type, Match and Match period (population 4 to 17 year old). **Table S2.** Gross and Per Capita Impressions of Cumulative Carlsberg Alibi Appearances by Type, Match and Match period (population 18 years and older). (DOCX 19 kb)
