# Supplementary material for: Carlsberg alibi marketing in the UEFA euro 2016 football finals: implications of Probably inappropriate alcohol advertising
Source: BMC Public Health. 2018 Apr 25;18:553. doi: 10.1186/s12889-018-5449-y (PMC5921309; doi:10.1186/s12889-018-5449-y)
Supplement: Supplementary file 1 — Table S1. Gross and Per Capita Impressions of Cumulative Carlsberg Alibi Appearances by Type, Match and Match period (population 4 to 17 year old). Table S2. Gross and Per Capita Impressions of Cumulative Carlsberg Alibi Appearances by Type, Match and Match period (population 18 years and older). (DOCX 19 kb) [file 12889_2018_5449_MOESM1_ESM.docx]

Table S1: Gross and Per Capita Impressions of Cumulative Alcohol Appearances by Type, Match and Match period (population 4 to 17 years old)

|  |  | Quarter final | | | | | | | | Semi final | | | | Final | |
| --- | --- | --- | --- | --- | --- | --- | --- | --- | --- | --- | --- | --- | --- | --- | --- |
|  |  | Poland v Portugal | | Wales v Belgium | | Germany v Italy | | France v Iceland | | Portugal v Wales | | Germany v France | | Portugal v France | |
|  |  | *Probably* | *The best in the world* | *Probably* | *The best in the world* | *Probably* | *The best in the world* | *Probably* | *The best in the world* | *Probably* | *The best in the world* | *Probably* | *The best in the world* | *Probably* | *The best in the world* |
| Gross Impressions (in millions) | End of 1^st^ half | 12.7 | 6.4 | 20.4 | 5.6 | 22.1 | 5.3 | 1.1 | 0.0 | 13.9 | 4.8 | 26.5 | 9.0 | 27.0 | 10.1 |
|  | End of 2^nd^ half | 33.3 | 12.2 | 53.8 | 13.0 | 53.4 | 14.5 | 6.9 | 0.0 | 31.0 | 10.7 | 64.3 | 20.1 | 74.4 | 19.2 |
|  | End of 1^st^ extra | 45.0 | 13.7 | - | - | 72.5 | 16.0 | - | - | - | - | - | - | 96.9 | 21.4 |
|  | End of 2^nd^ extra | 60.2 | 15.2 | - | - | 97.0 | 18.3 | - | - | - | - | - | - | 121.7 | 25.9 |
|  | End of penalties | - | - | - | - | 145.0 | 18.3 | - | - | - | - | - | - | - | - |
|  | Total (any alc.) | 75.4 | | 71.3 | | 163.3 | | 6.9 | | 41.7 | | 84.4 | | 147.6 | |
| Per Capita | End of 1^st^ half | 1.2 | 0.6 | 1.9 | 0.5 | 2.1 | 0.5 | 0.1 | 0.0 | 1.3 | 0.5 | 2.5 | 0.9 | 2.6 | 1.0 |
|  | End of 2^nd^ half | 3.2 | 1.2 | 5.1 | 1.2 | 5.1 | 1.4 | 0.7 | 0.0 | 2.9 | 1.0 | 6.1 | 1.9 | 7.0 | 1.8 |
|  | End of 1^st^ extra | 4.3 | 1.3 | - | - | 6.9 | 1.5 | - |  | - | - | - | - | 9.2 | 2.0 |
|  | End of 2^nd^ extra | 5.7 | 1.4 | - | - | 9.2 | 1.7 | - |  | - | - | - | - | 11.5 | 2.5 |
|  | End of penalties | - | - | - | - | 13.7 | 1.7 | - |  | - | - | - | - | - | - |
|  | Total (any alc.) | 7.1 | | 6.7 | | 15.5 | | 0.7 | | 3.9 | | 8.0 | | 14.0 | |

^*^ Calculations are based on Mid-year population estimates for the UK, which is 10.6 million people for over 4 to 17 years old.

Table S2: Gross and Per Capita Impressions of Cumulative Alcohol Appearances by Type, Match and Match period (population 18 years and older)^*^

|  |  | Quarter final | | | | | | | | Semi final | | | | Final | |
| --- | --- | --- | --- | --- | --- | --- | --- | --- | --- | --- | --- | --- | --- | --- | --- |
|  |  | Poland v Portugal | | Wales v Belgium | | Germany v Italy | | France v Iceland | | Portugal v Wales | | Germany v France | | Portugal v France | |
|  |  | *Probably* | *The best in the world* | *Probably* | *The best in the world* | *Probably* | *The best in the world* | *Probably* | *The best in the world* | *Probably* | *The best in the world* | *Probably* | *The best in the world* | *Probably* | *The best in the world* |
| Gross Impressions (in millions) | End of 1^st^ half | 168.8 | 81.3 | 250.7 | 68.4 | 292.3 | 68.2 | 14.2 | 0.0 | 196.8 | 65.5 | 279.3 | 93.1 | 308.5 | 115.7 |
|  | End of 2^nd^ half | 437.5 | 156.3 | 672.4 | 156.9 | 691.9 | 185.2 | 92.3 | 0.0 | 682.7 | 145.8 | 682.7 | 206.9 | 848.2 | 218.5 |
|  | End of 1^st^ extra | 593.8 | 175.0 | - | - | 945.3 | 204.6 | - | - | - | - | - | - | 1105.3 | 244.2 |
|  | End of 2^nd^ extra | 787.6 | 193.8 | - | - | 1257.1 | 233.9 | - | - | - | - | - | - | 1400.9 | 295.6 |
|  | End of penalties | - | - | - | - | 1880.8 | 233.9 | - | - | - | - | - | - | - | - |
|  | Total (any alc.) | 981.4 | | 829.3 | | 2114.7 | | 92.3 | | 828.5 | | 889.6 | | 1696.5 | |
| Per Capita | End of 1^st^ half | 2.7 | 1.3 | 4.1 | 1.1 | 4.7 | 1.1 | 0.2 | 0.0 | 4.5 | 1.1 | 4.5 | 1.5 | 5.0 | 1.9 |
|  | End of 2^nd^ half | 7.1 | 2.5 | 10.9 | 2.6 | 11.2 | 3.0 | 1.5 | 0.0 | 11.0 | 2.4 | 11.0 | 3.3 | 13.7 | 3.5 |
|  | End of 1^st^ extra | 9.6 | 2.8 | - | - | 15.3 | 3.3 | - | - | - | - | - | - | 17.9 | 3.9 |
|  | End of 2^nd^ extra | 12.7 | 3.1 | - | - | 20.3 | 3.8 | - | - | - | - | - | - | 22.6 | 4.8 |
|  | End of penalties | - | - | - | - | 30.4 | 3.8 | - | - | - | - | - | - | - | - |
|  | Total (any alc.) | 15.9 | | 13.4 | | 34.2 | | 1.5 | | 13.4 | | 14.4 | | 27.4 | |

^*^ Calculations are based on Mid-year population estimates for the UK, which is 61.9 million people for over 18 years old.
